# Direction of global health status: a narrative review and recommendation for incorporating integrative medicine for improvement

**DOI:** 10.25122/jml-2025-0155

**Published:** 2025-09

**Authors:** George Vithoulkas, Seema Mahesh

**Affiliations:** 1 University of the Aegean, Mytilene, Greece; 2 International Academy of Classical Homeopathy, Alonissos, Greece; 3 Centre for Classical Homeopathy, Bengaluru, India

**Keywords:** global health, health status, time trends, geographical trends, complementary and alternative medicine

## Abstract

Conventional medicine has made significant advancements in the past century, yet the global burden of chronic degenerative diseases has continued to rise impressively. Are we healthier than we were a hundred years ago? A narrative review focusing on the direction global health has taken under modern medicine was conducted using PubMed, Google Scholar, WHO, and CDC databases. The retrieved studies yielded additional historical data, which was also included. The bigger picture emerging from these sources is presented in a narrative form. Our review of historical and current medical literature suggests an alarming worsening of health status in the overall population, with a shift from infectious diseases to chronic debilitating conditions, including serious immune, neurological, and psychiatric illnesses. The countries with well-established healthcare systems are experiencing a higher burden of chronic degenerative diseases. It appears that the healthcare approach has focused on specific aspects rather than considering the complete picture of human health. We propose that healthcare innovations should refocus on studying the individual in their environment as an integral entity and conduct research to understand the long-term effects of medicines and vaccines. Further, integrating complementary and alternative medicine systems that consider health and disease holistically is recommended for incorporation into healthcare. However, it is emphasized that theoretical scientific research in this area remains limited, and there is a growing call for research in complementary medicine healthcare innovations, which, if executed well, may benefit living beings.

## Introduction

Medical science has advanced to the point where full organ replacement with robotic alternatives may soon become a reality. However, the need for such inventions was spurred by an increasing global burden of non-communicable and chronic diseases (NCDs) [1], bringing forth the question: *"After all these advances, are we really better in terms of overall health?"*.

To broaden the concept of health beyond the World Health Organization’s definition [2] Vithoulkas offers a more holistic perspective: *"Health is freedom from pain in the physical body, having attained a state of well-being; freedom from passion on the emotional level, having as a result a dynamic state of serenity and calm; and freedom from selfishness in the mental sphere, having as a result total unification with the objective truth."* [3].

Reflecting on where we stand today compared to just a few decades ago in relation to this ideal may help us better navigate the path forward.

## Material and Methods

A narrative review was conducted because the subject under consideration was vast and required examining historical descriptive records. The primary focus was the direction that global health has taken under modern medicine. Search was conducted under the following domains:

- Current global burden of disease (GBD) and mortality trends
- Historical GBD and mortality trends
- Medical coverage of different geographical areas and their disease trends
- Major pharmaceutical discoveries/usage and temporal trends of diseases
- Special populations – tribes/non-conventional medicine users and their disease trends

PubMed and Google Scholar were searched using various combinations of intervention- and population-related keywords, including: global burden of disease, population health, health trends, drug us*, medication us*, temporal, anthropogenic, exposure, antipyretic, analgesics, antibiotics, antidepressants, vaccin*, autoimmune diseases, neurological dis*, psychiatric dis*, mental health, and *delayed effects*. Additionally, screening the references of retrieved studies yielded further relevant historical sources that were not identified through the initial direct search.

Inclusion criteria for the studies required either a description of current or historical health statistics or a narrative account of health-related scenarios. Papers with geographical health status descriptions were also included. No time constraint was applied, and only English language papers were included. In addition, historical and geographical health trends were retrieved from the Center for Disease Control and Prevention (CDC) and World Health Organization (WHO) databases. A total of 94 sources were reviewed, and the overarching patterns identified are presented narratively in this work.

## The review

### Transformation of global health

Before the advent of modern pharmaceuticals, infectious diseases were the primary cause of death worldwide. Populations were heavily affected by infections, with high infant mortality rates and significantly shorter life expectancy [4]. In the early 1900s, the leading causes of death included respiratory infections, tuberculosis (TB), gastrointestinal infections, measles, diphtheria, typhoid, and syphilis.

Although antimicrobials began to reduce postoperative mortality by the late 19^th^ century, infectious diseases continued to dominate mortality statistics well into the early 20^th^ century [5]. TB remained widespread and highly lethal, while syphilis reached peak mortality during the 1930s. In the United States alone, syphilis caused approximately 20,000 deaths in 1939 [6]. In Australia, TB resulted in 10% of the deaths, and 10% of pregnant women tested positive for syphilis. Measles, diphtheria, gastroenteritis, and scarlet fever killed one in every 30 children born in 1911 in Australia [7].

The discovery of penicillin in 1928 and streptomycin in 1943 marked a turning point in infectious disease control, initiating what is often referred to as the golden era of antibiotic development between the 1950s and 1970s [6,8,9]. These breakthroughs led to a steep decline in infection-related mortality, particularly in industrialized nations. Life expectancy has risen by 29.2 years since the discovery of antibiotics and vaccines [10]. By 1997, only 4.5% of deaths in the US were attributable to infections [4]. In 2012, only 25% of the global deaths could be attributed to infectious diseases [11]. However, sexual liberation, fuelled by the invention of the birth control pill, resulted in abuse of antibiotics for syphilis and the rise of the more resilient and insidious infections such as gonorrhoea [7]. An explosive increase in the use of antibiotics occurred worldwide, pioneered by industrialized nations such as France, the USA, Spain, and New Zealand, and emulated by developing countries more recently [12].

Today, the top 10 causes of death globally are dominated by NCDs [11].

The analysis of CDC data on time trends for various diseases from 1900 to 1960 leads to the following general interpretations [4].

- Leading causes of death, which were infections in the 1900s, shifted to diseases of the heart, cancer, and cerebrovascular diseases by the 1940s, a pattern sustained even in the 1960s.
- A steep decline occurred in the mortality from TB, syphilis, dysentery, typhoid, diphtheria, and other infectious diseases between 1945 and 1960.
- The same period shows a gradual rise in the deaths from cardiovascular and renal diseases.
- Malignant neoplasms, which were moderate in their death rates in 1900, reached very high levels by 1960.

In the 19^th^ century, diabetes mellitus (DM) was insignificant. Johns Hopkins Hospital, USA, recorded only 10 out of the 35,000 patients admitted in 1892 as diagnosed with DM [13]. Today, 529 million people are living with DM, making up 6.1% of the global population [14].

In the early 1900s, mortality rates from type 1 diabetes mellitus (T1DM)—which was then universally fatal and thus directly reflected incidence—ranged between 1.3 and 3 per 100,000 in developed countries [15]. In contrast, by 2020, the global incidence had risen to 15 per 100,000 people, with a prevalence of 9.5% (95% CI, 0.07–0.12). Notably, nearly 49% of all cases were reported in developed countries, even though these nations account for only 17% of the global population [16]. Upon closer examination, the role of economic development and increased healthcare coverage seems to have a paradoxical effect on the health of the population, implying that indiscriminate use of drugs is a major factor in this transformation.

Low-income countries do not appear among the top 20 nations in terms of T1DM prevalence today [17,18]. A striking example of environmental influence comes from the neighboring populations of Finland and the Russian Karelia region, who share similar genetic susceptibility to T1DM. However, the Finnish population, with considerably better healthcare, showed a 6-fold increase in the incidence of T1DM compared to the Russian [19,20]. Similarly, children of immigrants to Sweden demonstrate a higher risk of developing T1DM than their counterparts in their countries of origin, despite originating from low-risk populations [21]. This sharp rise in incidence began in developed countries in the mid-19^th^ century, and a similar “catch-up” pattern is now emerging in developing nations as public health infrastructure and economic conditions improve [13,15]. For instance, hypertension and diabetes mellitus, once rare in sub-Saharan Africa, are now reaching endemic levels [22].

Compared to a few decades ago, a substantial proportion of the global population now suffers from autoimmune diseases, currently estimated at 11–21% [23]. According to the epidemiological transition model of Omron, this transition may be categorized as man-made diseases, as autoimmune diseases are primarily a bane of the industrialized world, with increased drug and vaccine usage [24]. A systematic review found a net increase of 19.1 ± 43.1% in their incidence per year in the last three decades. Rheumatic, endocrinological, gastrointestinal, and neurological autoimmune diseases increased by 7.1%, 6.3%, 6.2%, and 3.7%, respectively [23].

The prevalence of antinuclear antibodies (ANA), a nonspecific biomarker of autoimmunity, in the US population increased from 11% (95% CI, 9.7–12.6) between 1988 and 1998 to 16.1% (95% CI, 14.4–18.0) between 2011 and 2015 (*P* for trend < 0.0001), independent of obesity or smoking/alcohol consumption [25]. Rheumatoid arthritis incidence rose globally by 7.4% between 1990 and 2017, with the highest incidence and prevalence reported in the United Kingdom [26]. In Finland, considered to have one of the highest-quality healthcare systems, the incidence of celiac disease doubled by 2001 compared to the previous two decades [27]. Systemic lupus erythematosus (SLE), a major cause of mortality in women, has also shown a global rise, with prevalence estimated at 5.14 per 100,000 (95% CI, 1.4–15.13), particularly in high-income countries [28]. In the United States, SLE incidence increased from 3.32 per 100,000 (1976–1988) to 6.44 per 100,000 (2009–2018) [29].

**Figure 1:**
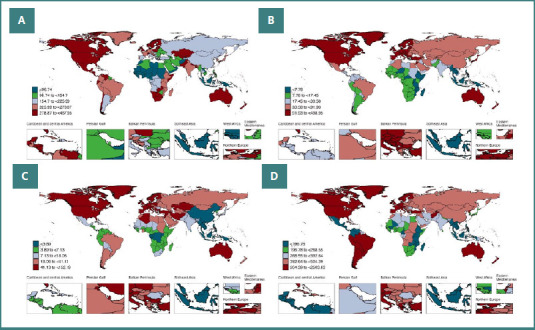
World map showing age standardized prevalence rates of (A) Rheumatoid Arthritis, (B) Inflammatory Bowel Diseases, (C) Multiple Sclerosis, and (D) Psoriasis.

**Figure 2:**
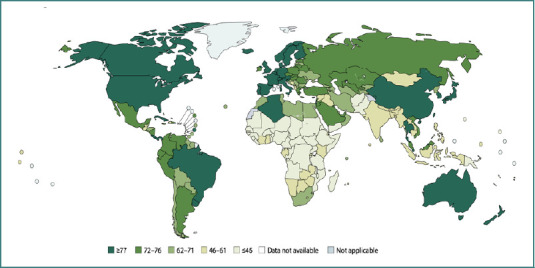
2017 Global healthcare coverage map

**Figure 3:**
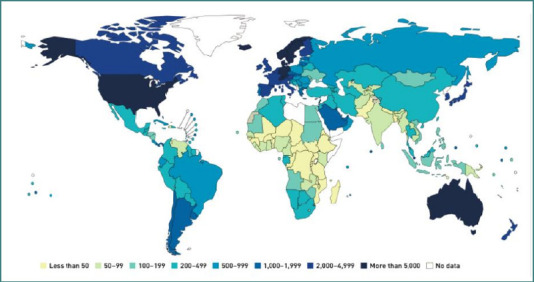
2017 Healthcare expenditure in US$

Figure 1 [30] shows the recent distribution pattern of four common autoimmune diseases. When these patterns are compared with global maps of healthcare expenditure and insurance coverage (Figures 2 and 3), a clear trend emerges: countries with higher medical expenditure and broader access to healthcare exhibit higher prevalence of autoimmune diseases [31,32].

In the BRICS countries (Brazil, Russia, India, China, and South Africa), rising economic development has been paralleled by a marked increase in autoimmune and inflammatory conditions such as psoriasis, multiple sclerosis (MS), SLE, and inflammatory bowel disease (IBD), with cases disproportionately concentrated in urban populations [33]. A similar trend is evident in sub-Saharan Africa, where the total Disability-Adjusted Life Years (DALYs) attributable to non-communicable diseases (NCDs) rose from 90.6 million in 1990 to 151.3 million in 2017—a 67% increase [34].

Today, however, we are dealing not just with relatively simple chronic diseases such as metabolic and endocrine disorders, but severe diseases characterized by neurodegeneration, immune impairment, and psychological disturbance [35-37].

**Figure 4:**
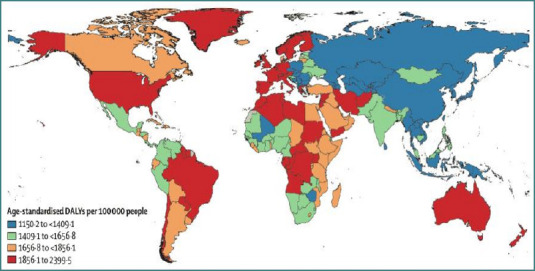
Map of the world showing disability adjusted life years for mental disorders

Historically, psychiatric illnesses were rare and often secondary to clear medical causes such as brain tumors or chronic alcoholism, with incidence remaining steady at approximately 1 per 1,000 individuals in Great Britain and the United States until the mid-19^th^ century [38]. However, between 1990 and 2019, the global burden of mental disorders increased from 80.8 million to 123 million DALYs [39], with anxiety disorders alone rising by 50% during this period [40]. Schizophrenia followed a similar trajectory, with prevalence increasing by 65% and incidence by 37.11%, particularly in high-income nations such as the United States and Australia [40,41]. As illustrated in Figure 4, psychiatric disorders demonstrate the same pattern observed with autoimmune diseases, showing higher prevalence in countries with greater healthcare expenditure and economic development [40]. For example, schizophrenia incidence is only 0.1% in Nigeria compared to 8.6% in Canada [40]. Likewise, mental health–related DALYs in India increased from 2% (95% CI 2.0–3.1) in 1990 to 4.7% (95% CI 3.7–5.6) in 2017 [42].

Autism prevalence rose from 4 to 5/10,000 births in 1996 to 14.9 to 34/10,000 births in recent times, not attributable to increased diagnosis alone [43]. Similarly, the global consumption of attention deficit hyperactivity disorder (ADHD) medications has increased by an estimated 9.72% annually (95% CI, +6.25%, +13.31%), with striking disparities between high- and low-income countries. In 2019, high-income countries reported a pooled consumption rate of 6.39 defined daily doses per 1,000 inhabitants per day (DDD/TID) (95% CI, 4.63–8.84), compared to only 0.02 DDD/TID (95% CI, 0.01–0.05) in lower-middle-income countries [44]. In the United States, adolescent psychotropic drug use increased two- to threefold between 1987 and 1996, a trend mirrored in Norway and Denmark [45]. Likewise, in 2018, the consumption of symptomatic dementia medications in high-income countries ranged from 3.88 to 5.04 DDD per 1,000 inhabitants per day, whereas in low-income countries it remained drastically lower at 0.094 to 0.396 DDD per 1,000 inhabitants per day [46].

Diseases like amyotrophic lateral sclerosis (ALS), MS, and Alzheimer’s disease (AD) are also becoming more common. The prevalence of MS increased from 24/100,000 in 1961 to 230/100,000 in 2006. Norway had the highest incidence, with no evident latitude gradient to account for the cold environment as a cause [47].

The incidence of Alzheimer’s disease increased nearly 40-fold between 1907 and 1911. By 2005, the diagnosis was occurring as frequently as one new case every seven seconds [48]. This was not attributable to increased longevity alone, as presenile cases increased as well [48]. AD or similar dementia was unknown in the developing countries until the 1980s. Hospital records from a Nigerian cohort of 350,000 individuals aged ≥65 years between 1957 and 1990 reported no cases at all. In contrast, genetically similar populations in the United States showed a substantial incidence—recent estimates place AD prevalence at 1.41% among Nigerians compared to 3.69% among African Americans [48,49]. A study comparing the microscopic slides of brain samples from India and the Western population before 1985 found no slides with “tangles” (characteristic of AD) in the Indian sample but found them in 15% of the Western population [48].

Even infections in recent times have morphed, from those meant for natural selection (epidemics) to those that are persistent/ low grade, leading to cancerous changes or neurological disorders.

This is especially so in the developing world, where the epidemiologic transition is still occurring from acute infections to NCDs. Hepatitis B (HBV), H pylori (HPy), and Human papilloma virus (HPV), the main infectious causes for cancers, are rampant in low- and mid-income countries. The developed countries plateaued out a few decades earlier and are currently in regression [50]. These infections, resulting from a lack of sanitation and hygiene, cause chronic organ damage. HPV is the most common sexually transmitted infection, with at least half of the people infected with the high-risk type of the virus that can cause cancer [51]. HPy, a bacterium that can cause gastric cancer, has a prevalence as high as 70% in some populations (~ 4.4 billion individuals). However, it must be noted that the African population, which shows the highest incidence, still has the lowest occurrence of cancer [52], indicating that the immune system is still preserved in this population.

Asia accounts for 75.3% of the DALYs from HBV, followed by Africa (11.55). The trend towards a decrease is attributed to extensive vaccination. However, the effect of the vaccination was evident only in children, and the decrease in adults had occurred even before the effect of universal vaccination could be seen [53]. Furthermore, investigation of HBV patients over three decades showed that recent patients tended to have less or no detectable viral load, but a higher risk for hepatocellular carcinoma, and more hepatic and renal comorbidities [54]. These phenomena demonstrate the change from preventable, easily treatable infections to deeper, more challenging-to-treat conditions.

### Role of pharmaceuticals in this shift

Pharmaceuticals played a major role in this transition. A 65% increase in the global use of antibiotics occurred between 2000 and 2015 [55]. As a result, despite being recognized in the early 70s, antibiotic resistance continues to plague the world today [12]. Resistant infections cause high mortality, and the fear that a significant number of infections may no longer be treatable is real [9,56]. In Africa, the eradication of Yaws with penicillin made the population more susceptible to syphilis [57]. A considerable decline in TB deaths occurred even before the widespread implementation of anti-tubercular treatment in the 1950s, attributed to public health measures of screening, sanitation, nutrition, and education [35], questioning the real role of these drugs.

The effect of antibiotics is far more reaching than just antibiotic resistance, as they interfere with healthy gut microbiota. Children exposed to antibiotics in the first year of life have a significantly higher risk of allergies, asthma, and obesity. Neurodevelopmental disorders such as ADHD and psychiatric disorders in genetically susceptible individuals are also attributed to such exposure [58]. Even a single exposure to antibiotics significantly increased the risk for depression and anxiety, [OR = 1.23 (1.18, 1.29) for penicillins, 1.25 (95% CI, 1.15–1.35) for quinolones], which further increased with repeat exposure [1.40 (1.34, 1.46) for penicillins and 1.56 (1.46, 1.65) for quinolones] [59]. Exposure of adolescent mice to antibiotics resulted in a gene expression alteration related to anxiety [58].

Such inadvertent effects are seen with other drugs as well. Miliary sclerosis of the brain (AD) rose significantly after the influenza pandemic in 1889 and the Spanish flu pandemic in 1918. Impairment of cognition was observed in phenacetin users, who extensively used phenacetin during these epidemics. However, developing nations, with limited access to conventional drugs, remained free of it [48].

The routine use of antipyretics to control fever during infections has been strongly questioned in the same vein [60]. A positive correlation and a dose dependency were found for exposure to acetaminophen and incidence of allergic rhinitis, rhino-conjunctivitis, asthma, and eczema [OR = 1.54 (1.41–1.69)] [61]. Autism risk seems to be associated with acetaminophen use, possibly attributed to oxidative stress and neurotoxicity generated by the drug, and to the blockage of fever during infections (interference with IL-6 release in infants, affecting normal brain development) [43, 62, 63].

One study observed that patients who later developed IBD had a progressive increase in healthcare utilization, specifically visits to emergency departments, general practitioners, and gastroenterologists, during the five years preceding diagnosis. Furthermore, Crohn’s disease (CD) and indeterminate colitis were associated with significantly higher prior use of antibiotics, nonsteroidal anti-inflammatory drugs (NSAIDs), proton pump inhibitors (PPIs), and etanercept [64].

Another major factor that has transformed modern healthcare is vaccination. Vaccines have eradicated certain infectious diseases [10] and substantially reduced the severity and incidence of many others. However, growing attention has been directed toward their potential long-term and subtle effects. While vaccines are designed to elicit protective immunity in all recipients, individual responses can vary widely due to differences in genetics, comorbidities, and other health-related factors [65].

The association between increased prevalence of allergies is dramatic in the age group usually subjected to national vaccination programs [66,67], but the most common negative effect from vaccination is the activation of autoimmunity. Guillain-Barré Syndrome, RA, SLE, MS, acute/chronic transverse myelitis, Behcet’s disease, Raynaud’s syndrome, ADHD, and autism are some of the diseases that have been associated with vaccinations [68,69].

Higher measles IgG titre (from MMR) was associated with brain autoantibodies, indicating an autoimmune induction phenomenon. In children who regressed after MMR, ileocolonoscopy found lymphonodular hypertrophy, a sign of chronic viral infection. This implies that MMR causes autism by inducing inflammatory bowel syndrome [70].

40% of parents of children with autism report that the child regressed after a vaccination [43]. Boys who received the hepatitis B vaccine (HBV) during the neonatal period had a threefold higher relative risk of autism compared with those vaccinated after the first month of life or not vaccinated [71]. A study conducted by the Vaccine Adverse Events Registration System (VAERS) found a significant odds ratio for developing different autoimmune and neurological diseases from HBV vaccination (Table 1) [72].

**Table 1:** Serious adverse events following HBV. Prospective case control study from VAERS:

Disease	OR (95%CI)	*P*
MS	5.2 (1.9, 20)	<0.003
Optic neuritis	14 (2.3, 560)	<0.0003
Vasculitis	2.6 (1.03, 8.7)	<0.04
Arthritis	2.01 (1.3, 3.1)	<0.0003
Alopecia	7.2 (3.2, 20)	<0.0001
Lupus	9.1 (2.3, 76)	<0.0001
RA	18 (3.1, 740)	<0.0001
Thrombocytopenia	2.3 (1.02, 6.2)	<0.04

This data is in comparison with tetanus vaccination. OR: odds ratio; *P* < 0.05 is considered significant

Similarly, another study found an OR of 3.1 (95% CI, 1.5–6.3) for the development of MS in people who received HBV vaccination three years before the index date, compared to those who had not [73].

An increased risk of Guillain–Barré syndrome (GBS) was also reported following the 1976 mass influenza vaccination campaign in the United States, where the incidence rose four- to eight-fold [74]. Additionally, an uptick in narcolepsy cases was observed in the Swedish and Finnish populations after administration of the AS03-adjuvanted H1N1 vaccine [65]. The HPV vaccine helped reduce the burden of cervical cancer significantly [75], and yet, it is associated with many chronic degenerative diseases. Quadrivalent HPV vaccine resulted in increased risk of Hashimoto's disease [76]. Another large study found the OR for SLE after vaccination to be 7.626 (95% CI, 3.385 – 19.366) with the onset in a median period of 3 to 37 days after vaccination [76]. Acute disseminated encephalitis and other demyelinating diseases of the central nervous system, such as MS and neuromyelitis optica, have been reported as occurring within a few days of the vaccine. Antiphospholipid syndrome, primary ovarian failure, autoimmune neuromyotonia, Henoch Schonlein purpura, cutaneous vasculitis, autoimmune hepatitis, Kikuchi-Fujimoto disease, cerebellar ataxia, erythema multiforme, immune-mediated thrombocytopenic purpura, linear IgA bullous dermatosis, and postural orthostatic tachycardia syndrome (POTS) have all been reported as resulting from HPV vaccination [76,77].

A recent study found that the current vaccine schedule required for school attendance may be associated with increased risk of neurodevelopmental disorders (NDDs). The study found that even one visit related to vaccination was associated with increased relative risk of autism spectrum disorder by 1.7 times. This was worse in preterm children, where 39.9% of vaccinated preterm children had NDDs as opposed to 15.7% of preterm unvaccinated children. The authors concluded that the current vaccine schedule may be contributing to multiple forms of NDDs and call for further study in this regard [78]. A study in the developed countries found that the number of neonatal vaccine doses required was positively correlated with neonatal mortality (r = 0.34, *P* = 0.017), infant mortality (r = 0.46, *P* = 0.008), and under-five mortality (r = 0.48, *P* = 0.004) [66, 67]. A significant difference was seen in just two doses of neonatal vaccines compared to no neonatal vaccine mandate (1.28/1000 live births, *P* < 0.002). Further, it was observed that vaccinated children had higher hospitalization rates than unvaccinated children. Sudden infant death syndrome (SIDS) has been associated with vaccination (of different types) [66,67].

Several studies have described instances of *de novo* autoimmune or inflammatory conditions following COVID-19 vaccination. These have included Graves’ disease, rheumatoid arthritis, palindromic rheumatism, adult-onset Still’s disease, polyarteritis nodosa, systemic lupus erythematosus, polymyalgia rheumatica, autoimmune thrombocytopenia, Guillain–Barré syndrome (GBS), IgA nephropathy, Bell’s palsy, seizures, acute and chronic transverse myelitis, acute disseminated encephalomyelitis, cerebral venous sinus thrombosis, stroke, and others [68,69,79]. A recent study reported a significant increase in the cumulative incidence of depression, anxiety, dissociative and stress-related disorders, somatoform conditions, and sleep disturbances following COVID-19 vaccination [80]. The observed-to-expected ratios were calculated for 13 conditions in a very large cohort of COVID-19 vaccinations. GBS, acute disseminated encephalitis, myocarditis, and pericarditis showed a significant increase compared to expected adverse events following the mRNA and adenovirus vector vaccinations in this study [81]. All these diseases compromise, if not severely debilitate, the quality of life.

Areas in rural India, where people are wary of conventional medicine, provide interesting observations. The Amarakantak region is known for its traditional herbs, and people do not commonly avail themselves of conventional care. NCDs have increased here too, but are mostly simple chronic diseases (backache, musculoskeletal issues), undernutrition, and alcoholism. In terms of autoimmune, metabolic, cardiovascular, renal, and other similar diseases, this population is healthier than its neighbouring urban dwellers [82]. This is true of most tribal communities in India. A third of the world’s indigenous people live in India, and they mostly practice herbal/alternative medicine. With 41% literacy and 56% vaccination coverage, their usage of public health services is basic at best. These people still suffer infections and do not add to the global NCDs burden [83]. Raikas, a camel herding tribe in western India, has the diabetic gene similar to the rest of the population of that region, yet has not a single documented case of T1DM. This tribe depends on the camels for their livelihood, uses indigenous herbal medicine, and does not access conventional medicine/public health services like its fellow citizens [84].

Native Americans continue to experience higher rates of infectious diseases, such as tuberculosis, compared to the general U.S. population [85]. Children in farming communities such as the Amish, where traditional methods of farming have continued, have a significantly lower prevalence of asthma compared to the non-Amish children. While the exposure to cows, straw, fodder, animal manure, and unprocessed milk has a protective effect against allergies, the Amish also prefer complementary and alternative medicine over conventional medicine. Only 45% of Amish children are vaccinated, and over 59% of their population refuses any kind of vaccination [86,87].

In Australia, Aboriginal children show an increased burden from upper respiratory tract infections, scabies, and skin sores, and are more likely to be hospitalized from infections compared to the non-Aboriginal children [88]. Their public health service usage is also low.

These data demonstrate that populations that do not use conventional medicine primarily are still suffering from preventable infectious diseases and are less affected by NCDs.

### Likely mechanisms responsible for driving diseases deeper

**Figure 5:**
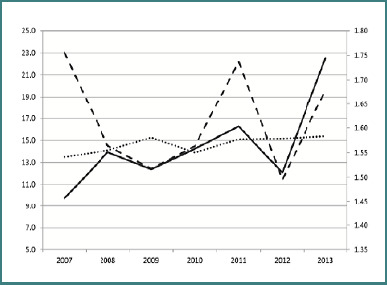
Schematic representation of the temporal association of T1DM among children aged 0–4 years (for 100,000 inhabitants, solid line), sales of paracetamol (DDD/1000/day, mono doses 80–300mg, dashed line) – right axis scale, milk consumption (kg per capita, dotted line, scaled down 20 times) – left axis scale, in Lithuania 2007 – 2013

Many studies have shown that the above phenomena are not merely temporal associations but occur due to conventional medical approaches. The induction of autoimmunity after an infection was determined to result from a combination of genetic predisposition to autoimmunity and the inflammatory response in which paracetamol plays a potentially mediating role by inhibiting cyclooxygenase enzymes, thereby inhibiting prostaglandin synthesis. In Lithuania, the temporal association between paracetamol sales and incidence of T1DM was observed, with the authors also suggesting dairy intake as a possible cofactor (Figure 5) [89].

Certain infections protect against allergies by triggering a Th1 lymphocyte response (an efficient inflammatory response) and prevent Th2 (typical of chronic inflammation) [90]. Therefore, it logically follows that if the acute inflammatory response is hindered, then the protection from chronic inflammatory conditions cannot be facilitated.

Infection with hepatitis A, RSV, S typhi, BCG, and other common infectious bacteria showed a significant protective effect against the development of allergies and autoimmune diseases [90]. However, these findings are equivocal as infections are known to induce autoimmunity as well [90].

There seems to be a delicate interplay between host and pathogen, genetics and immune systems, that decides the outcome.

The “Theory of a continuum of diseases” renders an integral explanation to this phenomenon [91]. It states that diseases form a continuum throughout a person’s life. While basic health is determined by genetics and parental psychological state, it is later influenced by lifestyle, the therapies adopted for diseases, and the stress one experiences. The role of therapeutic agents is significant in this context. The suppression of acute infectious diseases, especially, leads to a state of sub-acute inflammation, eventually triggering the chronic inflammatory disease to which one is predisposed. The same happens when the immune system is stressed with vaccinations. This theory finds support in other theories and established evidence. Immunological studies have demonstrated that interrupting the initial part of an acute inflammatory process dysregulates the downstream resolution of inflammation, perpetuating a low-grade inflamed state in the tissue and eventually triggering chronic inflammation [92].

Further, alteration of the gut microbiota (dysbiosis), as may happen with chemical drugs, leads to many chronic inflammatory conditions and neuro-psychological conditions through the gut-endocrino-immune-brain axis [36,93,94]. This lends strength to the argument that global gut health has been altered tremendously by the drugs in question, leading to a pandemic of NCDs and neuropsychological disorders.

### Why the indifference from medical research regarding this?

Many investigators have questioned the calm attitude of medical researchers regarding the long-term and cumulative effects of the pharmaceuticals on the global population. There is a dearth of studies examining such effects of drugs used for acute events [95]. The indifference probably stems from a combination of psychological, professional, and systemic factors. Usually, the priority of immediate patient care often takes precedence over longer-term considerations [96, 97]. However, the primary reason for such oversight lies in the psychology of medical practitioners. Although long-term side effects are well-documented and detailed in the accompanying drug information leaflets, doctors often hesitate to address these issues openly, not only due to the potential sensitivity for pharmaceutical companies but also because of their own professional roles as prescribers. Acknowledging and discussing these effects can place medical professionals in a challenging position, as it may inadvertently call their prescribing decisions into question, potentially undermining their credibility and patient trust. Consequently, it becomes a complex issue of balancing the immediate need to treat acute diseases with the responsibility to consider and communicate long-term risks.

Furthermore, the focus of conventional medicine over the century has become increasingly narrow, with researchers’ views also narrowing in the process. The whole organism, the bigger picture, has long been forgotten. A clinical example is the management of congestive cardiac failure, where treatment with renin–angiotensin–aldosterone system inhibitors and diuretics is often initiated with limited consideration for potential adverse effects on renal function. There are not even sufficient studies to provide evidence-based guidelines on how to manage the whole picture while such drugs are induced [98,99]. Such disconnect and fragmentation of the human organism lead to similar research, and they cannot see the bigger picture of the deteriorating global health.

### Summary

**Figure 6:**
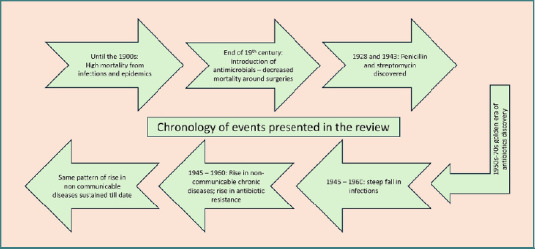
Chronology of events presented in the review

A timeline of events described in the review is shown in Figure 6. So far, our review arrives at the following interpretations:

- All medical drugs have side effects that appear immediately after their injection into the living organism.
- Research shows that in a great portion of the population, these interventions can create a serious compromise of the immune system.
- There is a discernible trend towards decline in the overall health of the global population, particularly in affluent countries. Chronic conditions are transitioning from predominantly affecting the physical body to having a pronounced impact on the mental, emotional, and spiritual well-being of the population.
- The major cause for this degradation is modern medical therapeutics, including drugs, antibiotics, and vaccinations.
- The importance of acute inflammatory response has been neglected, with a tendency to suppress it using potent pharmacological agents.

Thus, despite well-intentioned efforts and apparent victories in isolated battles, the healthcare system may be losing the bigger war in the long term. We have achieved extended lifespans at the cost of quality of life. There is an increasing dependence on medical technology for solutions. We have transitioned from a time when preventable infectious diseases and epidemics were a primary concern to a time when infections have become so formidable that neither sanitation measures nor antibiotics can effectively combat them. In our apprehension of infections, we neglect the gravity of chronic conditions, willingly accepting the daily intake of numerous pills to manage debilitating health issues.

External hormone supplementation is pursued without consideration for negative feedback mechanisms, leading to inadvertent suppression of natural glandular production. The suppression of the immune system through anti-inflammatory drugs and immunosuppressants renders the body susceptible to dangerous infections, even those resistant to potent antibiotics. The nuanced effects of vaccinations on the immune system, inducing allergies and autoimmunity, have been disregarded in our zealous pursuit of eradicating infections. The delicate equilibrium between host and pathogen has been neglected, replaced by a focus on pathogen elimination through force in the name of evidence-based medicine. In light of our initial definition of ideal health, we are drifting farther away from the concept of "freedom" on all levels.

### Recommendation for improvement

Through this review, it becomes apparent that modern healthcare has overlooked the broader context of the constitution, hyper-focusing on isolated components of living organisms. Does this necessitate a cessation of medical progress? On the contrary, there exists vast untapped potential for therapeutic development.

The primary recommendation is a paradigm shift. The research focuses on extremely minute details of specific pathways, factors, and genes, which is helpful, but we also need a broader investigation to progress in global health care. The authors advocate for a holistic approach that considers human beings as integrated entities within their environment. Precision and individualization in medicine could emphasize the nurturing of the immune system rather than solely addressing specific diseases. For example, it is now known that the pro-inflammatory process is essential for efficient anti-inflammation [92]. Research focusing on therapies that promote the efficiency of inflammation rather than suppress it can help the population’s overall health [100]. Therapies (including the holistic alternative and complementary) that triangulate the influence of hereditary predisposition, psychosocial stress, and environmental factors (including medications) must be funded and promoted by institutions for cutting-edge research that will benefit overall health. Further, long-term, meticulous research is essential to document the precise ways in which pharmaceuticals and vaccines affect health and the factors that affect their efficiency and benefit.

In medical education, doctors must be taught alternative systems as part of the curriculum with standardized, credible courses that make them sensitive to a holistic approach and focus on the root cause rather than the symptoms of disease. Doctors will then be open to non-drug alternatives where it is proven, helping to reduce the global burden of NCDs.

For the patient, it is necessary to enforce stricter rules regarding over-the-counter medication and to encourage addressing minor ailments with well-trained alternative therapists and healthcare workers rather than medical professionals. Ultimately, an ideal healthcare approach that prioritizes health protection over reactive symptom management may be achieved.

## Conclusion

This review sought to explore the overall global health status before and after the advent of modern pharmaceutical advancements to ascertain if we are heading in the right direction. While we have overcome the battle with infectious diseases to a great degree, a worse war is currently being waged against the increasing burden of chronic, sub-inflammatory, and degenerative diseases that have left a great proportion of the population dependent on pharmaceuticals/medical technology for everyday existence. The infections that affect us today are more serious and cannot be eradicated as easily with antibiotics and vaccines. The authors suggest a paradigm shift in medical research, education, and policy, towards integrating alternative and complementary therapeutic approaches, so that a higher standard of care, with a focus on health protection and individualized treatment, may follow.

The whole intellectual framework of the paper represents the ideas, experience, and vision of the author, Prof. George Vithoulkas
